# Current Status of Measurement Accuracy for Total Hemoglobin Concentration in the Clinical Context

**DOI:** 10.3390/bios12121147

**Published:** 2022-12-08

**Authors:** Elena Stawschenko, Tim Schaller, Benjamin Kern, Berit Bode, Frank Dörries, Kristina Kusche-Vihrog, Hartmut Gehring, Philipp Wegerich

**Affiliations:** 1 Department of Anaesthesiology and Intensive Care Medicine, University Medical Center Schleswig-Holstein, Campus Luebeck, 23538 Luebeck, Germany; 2 Institute of Biomedical Engineering, University of Luebeck, 23562 Luebeck, Germany; 3 Medical Sensors and Devices Laboratory, Lübeck University of Applied Sciences, 23562 Luebeck, Germany; 4 Northern Scientific Tec & Integration GmbH, Kollaustr. 11-13, 22525 Hamburg, Germany; 5 Institute of Physiology, University of Luebeck, 23562 Luebeck, Germany

**Keywords:** hemoglobin, accuracy, concentration, tolerance level analysis, patient blood management

## Abstract

Objective: The main objective of this investigation is to provide data about the accuracy of total hemoglobin concentration measurements with respect to clinical settings, and to devices within the categories of point-of-care and reference systems. In particular, tolerance of hemoglobin concentrations below 9 g/dL that have become common in clinical practice today determines the need to demonstrate the limits of measurement accuracy in patient care. Methods: Samples extracted from six units of heparinized human blood with total hemoglobin concentrations ranging from 3 to 18 g/dL were assigned to the test devices in a random order. The pool of test devices comprised blood gas analyzers, an automatic hematology analyzer, a laboratory reference method, and the point-of-care system HemoCue. To reduce the pre-analytic error, each sample was measured three times. Due to the characteristics of the tested devices and methods, we selected the mean values of the data from all these devices, measured at the corresponding total hemoglobin concentrations, as the reference. Main results: The measurement results of the test devices overlap within strict limits (R^2^ = 0.999). Only the detailed analysis provides information about minor but systematic deviations. In the group of clinically relevant devices, which are involved in patient blood management decisions, the relative differences were within the limit of +/− 5 % for values down to 3 g/dL. Conclusions: A clinically relevant change of +/− 0.5 g/dL of total hemoglobin concentration can be detected with all selected devices and methods. Compliance with more stringent definitions—these are the relative differences of 5 % in relation to the corresponding reference values and the clinically adapted thresholds in the format of a tolerance level analysis—was achieved by the clinical devices assessed here.

## 1. Background

Knowing the exact hemoglobin concentration is an essential part of perioperative patient care and determines the required strategy for patient blood management [1,2,3]. The accuracy of the total hemoglobin concentration (tHb) measurement (tHb in g/dL) is a fundamental requirement for decision-making. Red blood cell transfusion is associated with significant risks, and the number of blood donors is declining [4,5,6,7]. With adequate monitoring, significantly lower tHb levels can be tolerated clinically [2,8]. While in the past, levels below 8 g/dL regularly led to transfusions [8], levels as low as 6 g/dL are now tolerated in otherwise healthy and young patients, or if specific organ monitoring is undertaken [2,8]. In certain case reports, patients even survived Hb-levels below 4 g/dL without organ damage, albeit with close monitoring and supportive care, but without any transfusion of blood components [2]. All of this merely substantiates the need for a clearly adapted measurement accuracy of tHb within this range.

This investigation was borne by the need to assess the measurement accuracy of reference-type devices that are used for decision-making in patient blood management. These essentially constitute the latest generation of blood gas analyzers (BGA) as well as an automatic hematology analyzer for determining blood counts. Data from these reference devices and methods are also provided for calibrating non-invasive monitoring systems for tHb, including point-of-care testing (POCT) systems and monitoring (POCM) devices [9,10]. Such devices are also essential for comparing a wide range of investigations and tests on the subject of measuring hemoglobin concentration [11,12,13,14,15,16,17,18,19,20]. To complement and compare this collection of test procedures, we also included the alkaline haematin detergent method (AHD) as a manually assessed laboratory reference procedure [21,22], the HemoCue system as an established POCT method, and the earlier generation OSM 3 CO-oximeter device.

The aim of this investigation was to determine the measurement accuracy of the selected test devices and methods over the entire clinically represented range of 3–18 g/dL under standardized conditions. This meant that each device acquired its sample from the same blood unit with a defined tHb and with an assignment of the samples to the devices in a randomized order. In clinical application, a confidence level of +/− 0.5 g/dL for the measurement accuracy is targeted [23]. For devices of a reference character, these requirements should be at least identical, if not stricter. This applies over the entire measuring range and even more so to ranges of 3–9 g/dL. As instrument candidates, the methods of the relative differences in relation to the corresponding reference values within the limits < +/− 5% and the tolerance level analysis (TLA) [7] meet the requirements for testing with respect to these adjusted limits.

## 2. Material and Methods

### 2.1. Measurement Systems and Application

The test protocol is designed to resemble the clinical workflow as closely as possible. This includes measurements with human blood, the use of clinical standard syringes or cuvettes, and the implementation of measurements on devices used in clinical practice, although the AHD method as a reference laboratory standard method and the OSM 3 as an earlier generation CO-oximeter were also added to the protocol (Table 1).

Overall, essential steps had to be introduced to keep the pre-analytical error as low as possible. Since the procedure was designed to resemble clinical routine as closely as possible, however, this could only be done within limits. To avoid or reduce any pre-analytical errors, the following steps were included:

Sample preparation in the immediate vicinity in terms of time and space, standardized filling of the cuvettes, continuous agitation to prevent sedimentation of the erythrocytes, and three repeat measurements for each analysis.

In the case of two identical devices, each was provided three times with the same sample and in a randomized order to identify any systematic deviations. The test for the HemoCue included three devices.

### 2.2. Blood Gas Analyzer (BGA)

The BGA devices (GEM 4000, GEM 5000, ABL 80 FLEX CO-OX) are standard systems in this day and age. The measurement of hemoglobin concentration is carried out by the CO-oximetry module. These are so-called cartridge systems. This means that the sensor technology and the necessary solutions are installed in a cartridge format and are tested and made available for a specified period of time or a specified number of samples. An external calibration of the sensors is necessary for the GEM 4000 device. For the ABL 80 FLEX CO-OX and GEM 5000 devices, this is automatically integrated within the solution packs. In addition, all of these devices are subject to the strict guidelines of the German Medical Association for clinical use, and have an internal device quality management system [24]. Furthermore, these devices are controlled via the clinic’s network and are subject to regular checks by the central laboratory.

The OSM 3 is an earlier generation CO-oximeter, which was also a common reference method for testing pulse oximeters and measuring hemoglobin concentration. By involving it in this study, we also wanted to provide an effective comparison with historical CO-oximeter studies.

### 2.3. HemoCue 201^+^

HemoCue Hb 201^+^ [25,26] is a rapid and easy-to-use POCT system for determining total hemoglobin in whole blood using a microcuvette. The microcuvette contains the reagents in dry form and combines the functions of a pipette, a test tube and a volumetric microcuvette. The hemoglobin bound in the erythrocytes is released by sodium deoxycholate and converted into methemoglobin using sodium nitrite, which then reacts with sodium azide to form azidemethemoglobin. The absorbance is measured at two wavelengths (570 and 880 nm) to compensate for any turbidity. This system also has an internal quality control where an automatic self-test is carried out when the device is switched on and after intervals of 2 h during operation.

### 2.4. Sysmex XN 9000

This device is one of the family of so-called “automatic hematology analyzers” (AHA) and functions according to the principles of hydrodynamic focusing for counting the erythrocytes and platelets, flow cytometry for the physiological and chemical characterization of cells and other biological particles, and the sodium lauryl sulfate (SLS) method for measuring hemoglobin concentrations [27,28].

SLS is a low toxicity compound that forms an SLS hemoglobin complex by disrupting the erythrocytic membrane and producing SLS methemoglobin without an oxidative agent; it is suitable in this form for manual, spectrophotometric, and automated analytical methods. The SLS method is therefore rapid to implement, includes all forms of hemoglobin, and results in a solid and easily measurable complex. No toxic substances are produced during the reaction steps. These advantages permit an automated procedure for measuring hemoglobin concentrations in laboratory analyzers.

### 2.5. AHD Method

The alkaline hematin method (AHD method) is presented elsewhere in detail [29,30]. In principle, hemoglobin forms a color complex with the AHD reagent, whose absorbance is measured photometrically at 575 nm. The color intensity is proportional to the hemoglobin concentration. All clinically relevant hemoglobin variants including carboxyhemoglobin, sulfhemoglobin, and fetal hemoglobin are collectively recorded. The AHD method was introduced in the German Standard DIN 58931 for the first time in 2010 [21] in parallel with the hemiglobin cyanide method (HiCN) as a reference procedure. The use of a reagent solution that can be stored at room temperature, the principle of the optical measurement, and the classification as a reference method in [3,21,22] led to its application in this study. This method is not suitable for automatic analysis, since a reaction time of at least 5 min must be observed.

### 2.6. Preparation of Basic Blood Units

**Figure 1:**
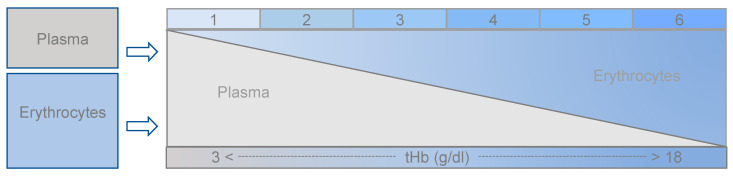
Configuration of the 6 basic blood units (approx. 20 mL, fast check with HemoCue), then separated in: 8 BGA cuvettes with 2 mL (Sarstedt, ∑ 16 mL); 2 blood count cuvettes with 1 mL (Sarstedt, ∑ 2 mL), 2 cuvettes with 1 mL (∑ 2 mL) for AHD processes, ABL 80, and OSM 3 HemoCue = 9 × microcuvettes.

The investigation was conducted in accordance with the Declaration of Helsinki Ethical Principles and Good Clinical Practices, and was approved by the local ethics committee of the University of Lübeck (registration number 20-058). After obtaining informed consent, 120 mL of blood was taken from an autologous blood donation in two infusion pump syringes (2 × 60 mL, anticoagulation with 500 IU heparin per syringe). Plasma and erythrocytes were prepared by gentle centrifugation and removed separately. Subsequently, plasma and erythrocytes were then combined into 6 units with tHb levels of approx. 3–18 g/dL (Figure 1). The samples for each test device were extracted from these approximately 20 mL basic units. Each of these 20 mL samples was immediately distributed to the test devices and systems using standard cuvettes. The samples were stored and transported with constant machine-assisted and manual rotation.

### 2.7. Data Acquisition

It should be noted that (1) each device received the blood samples from the corresponding basic blood units, and that (2) there was no absolute accurate reference value for the true tHb.

The test period extended over 3 identically structured laboratory days involving blood sampling and preparation of the basic units, leading to 198 measurements per day (see Table 1, 11 devices and methods with 3 measurements each at 6 defined tHb), resulting in a total number of 594 data points for further statistical processing.

### 2.8. Definition of References

In accordance with the initial situation that the systems tested here had already served as reference procedures in test studies elsewhere (summarized in [3] and [31]), and in accordance with Bland and Altman’s assumption [32,33,34,35,36,37,38] that each procedure has its own small but systematic error, we considered the mean of all data from the devices in the test as the best-fit correct target values for reference (REF).

According to the following steps, two REF data sets apply (Table 2). For step one, these are the mean values of all devices and procedures undergoing testing (REF I). For step two, these are the mean values of the clinically relevant devices and procedures (REF II).

The first step was the evaluation of the data of the respective individual devices against the defined target values (REF I) by means of regression analyses according to Deming and according to Passing–Bablok (PB). The root means square error (RMSE) and the R^2^ value (RSQ) ensured the accuracy of the data as quality criteria (Table 3). For further analysis, the data from structurally identical devices (HemoCue, GEM 4000, and GEM 5000) were averaged together.

The second step was to look at the data only from devices used in clinical patient care for making blood transfusion decisions. These are represented by the current generation BGA devices (GEM 4000, GEM 5000, and ABL 80) and the automated laboratory device (XN 9000).

### 2.9. Bland and Altman Method (B and A) and Prediction Interval

The graphical representations of the mean values +/− 1.96 SD according to the B and A method as bias and limits of agreement include the absolute (in g/dL) and the relative differences (in %) compared to the respective target values in (g/dL).

The prediction interval is an estimate of an interval within which a future tHb value will lie with a 95% probability, based on the available data from the study.

### 2.10. Tolerance Level Analysis (TLA)

In contrast to the error grid representation of Clark [16,39] we selected the TLA as representative due to the obvious advantages of this method. In particular, these take into account tHb values below 6 g/dL and the consideration of potential systematic errors regarding the measurement methods [7]. As with the error grid representation, representative zones are defined. In the TLA these are zones A–E: Zone A represents the requirements for detecting anemia, zone C is defined by the accuracy requirements for patent blood management, zone E is defined as the critically low area requiring the most accurate measurements. Zones B and D represent a seamless transition between these critical zones. The definition of zone E in the TLA represents the necessary and strict limits for levels of tHb below 6 g/dL, even if this is not demarcated in the error grid analysis.

## 3. Results

**Figure 2:**
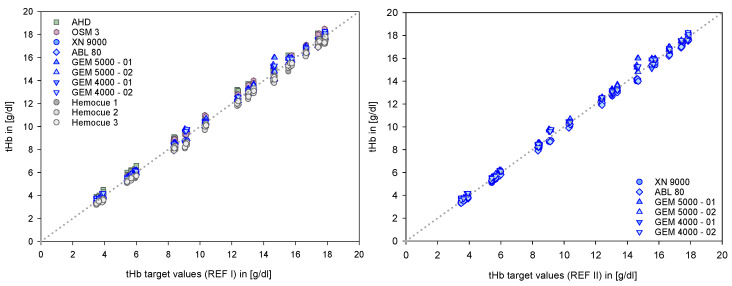
Data points of each device are plotted against their target values (mean of all). The left side shows the data from all devices in the test with REF I, the right side the data from the clinically relevant devices with REF II.

The primary data set contained n = 594 data points (see also Table 2). The comparisons of this data using regression analysis demonstrated the strong consistency of the selected devices, systems, and methods with the reference values (Figure 2), underlined by the quality criteria (Table 3).

**Figure 3:**
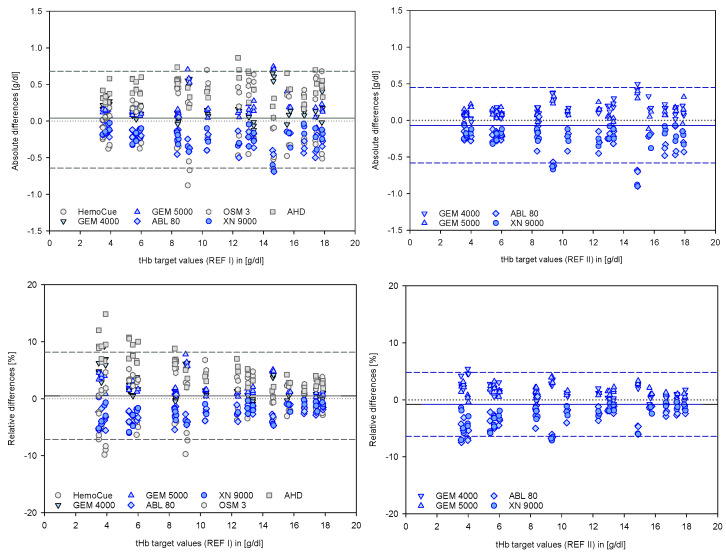
Absolute differences in g/dL (**top**) and relative differences in percent versus the respective target values (**bottom**) in g/dL. **Left**: for all devices in test with REF I, **right**: for the clinically relevant devices under test with REF II. Lines represent mean +/− 1.96 SD with respect to the B and A analysis.

For the purpose of further analysis, the data from the identical devices and systems are summarized by averaging. The plots of absolute and relative differences (Figure 3) highlight the small but significant variances among the devices, systems, and procedures undergoing testing. In particular, the details of the relative difference (in %) compared to the respective reference value filter out the critical deviations, especially in the low ranges. It is all the more gratifying that the clinically relevant devices do not reflect this effect.

The B and A procedure was introduced for comparing two methods or systems in medicine. The values of the absolute differences are presented in Table 4 and demonstrate small but systematic deviations. In the present study, we also used this method for assessing relative differences in order to ensure comparability here as well.

**Figure 4:**
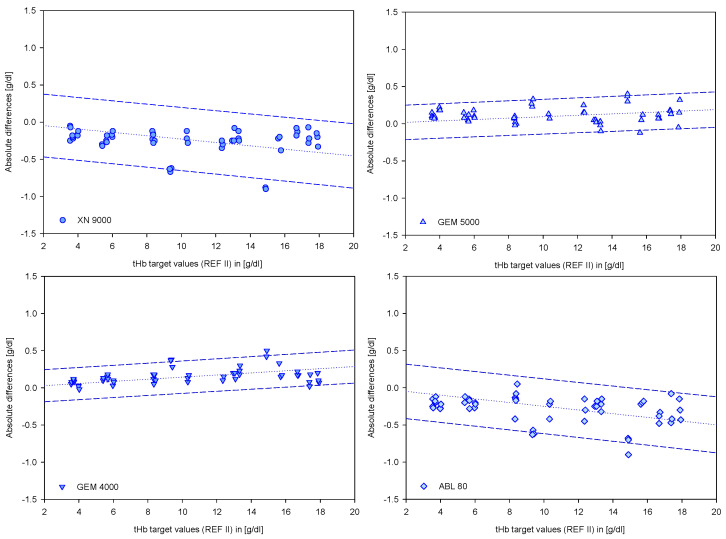
Prediction regression lines with the 95% prediction intervals of the clinically relevant devices. The graphs show data points from the clinically relevant devices versus target values (REF II).

**Figure 5:**
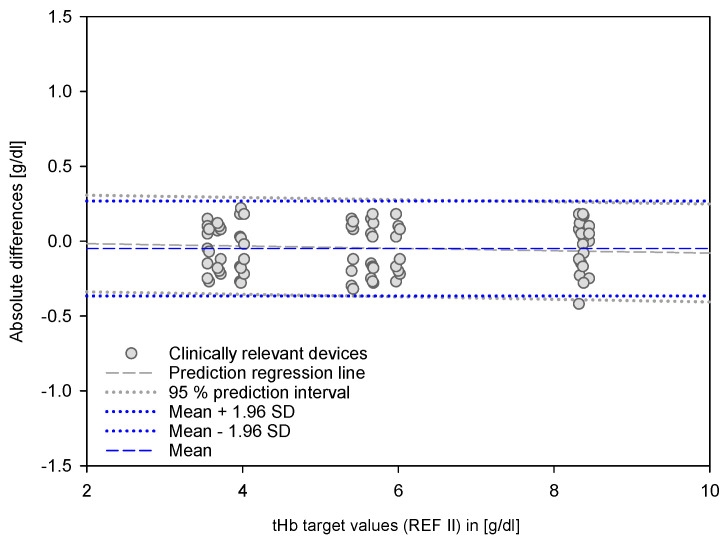
Absolute differences for the tHb segment below 9 g/dL for the clinically relevant devices against target values (REF II). The graph shows overlapping of B and A definitions with the 95% prediction interval.

For the clinically relevant devices, the 95% prediction interval for the differences provided the highest resolution in terms of measurement accuracy (Figure 4). According to the regression lines, there are small but significant linear dependencies on the variable being measured. Focused on the segment below 9 g/dL, it forms a cluster in which a linear relationship can be assumed. Independent of the device, this cluster has an average value close to zero and within the 95% interval at +/− 0.3 g/dL. The validity of this statement is verified by overlaying the B and A definitions with the prediction interval data for this range (Figure 5).

**Figure 6:**
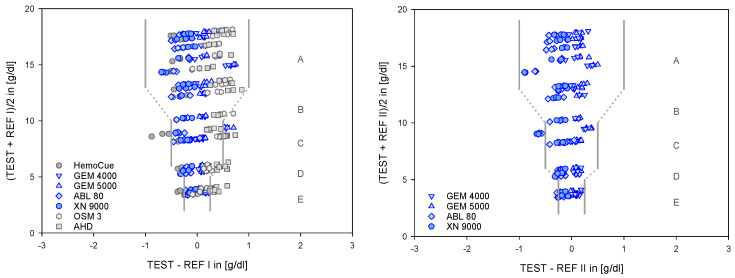
Data plot according to the tolerance level analysis (TLA). The measurement accuracy should be within the limits shown. Zone A represents the requirements for detecting anemia, zone C is defined by the accuracy requirements for patent blood management, zone E is defined as the critically low area requiring the most accurate measurements. Zones B and D represent a seamless transition between the critical zones, according to [7].

In addition, the mapping of the data within the strict limits required by the TLA and specially designed for the critical and low range of tHb under clinical observation almost confirms the compliance of the clinically relevant devices with the requirements (Figure 6).

## 4. Discussion

“Accurate diagnosis at the individual level is important to identify individuals who require treatment”. With this quote, Karakochuk [3] and his coauthors clearly formulated the needs underlying the objectives of this study from a clinical perspective.

A substantial goal of this study was to establish confidence levels in forms of limits of agreement and prediction intervals that a physician can rely upon in order to make safe decisions. The previously established clinical expectation that the maximum scatter of the measured values should lie within the limits of +/− 0.5 g/dL [23] is no longer sufficiently tenable for the current practice of tolerating extremely low tHb.

At this point, it is crucial for clinical devices (from which clinical decisions are derived) to have accuracies adapted to the segment of significantly reduced tHb levels as low as 3 g/dL. The BGAs and the automatic laboratory analyzer from the current generation have succeeded in doing this.

### 4.1. Limitations

#### 4.1.1. Definition of the References

This point can be addressed more precisely with a question: Which method is an absolute reference for hemoglobin? From today’s perspective, this can no longer be answered unequivocally. Because of the systematic limitations for each mode of measurement, we have to distinguish between manually and automatically implemented procedures. It needs to be emphasized that it is the automatic analyzers which are relevant for making clinical decisions.

#### 4.1.2. Manual Procedures from the Laboratory

The HiCN method has been described elsewhere in detail [23]. Its implementation in the laboratory requires intensive experience as well as the execution of manual steps. Some of the substances used here—especially potassium cyanide—are extremely toxic. In the study of 2002, a small but systematic difference could be determined between the HiCN and the AHA methods. In contrast to HiCN, the AHD method includes significantly fewer manual steps and is reduced to the use of just one substance. The procedure is equated with the HiCN method as a reference [21,22] and—given its advantages—was used here in the present study. With regard to its toxicity, the substance AHD 575 was classified as non-hazardous in 2010. However, this changed with the classification of the substance Triton X-100 for the lysis of erythrocytes as toxic in the Candidate List of Substances of Very High Concern in 2019 [40].

#### 4.1.3. Automatic Procedures

In principle, a distinction must be made between CO-oximetry as a module of a BGA and the SLS method of the AHA. In 2002, our working group was able to demonstrate small but systematic differences between the AHA and the HiCN method [23]. In the case of the CO-oximeters, there are also small but systematic differences between identical devices within a series and company on the one hand, and between individual companies on the other [31].

Generally, the assumption of the B and A procedure, that a small but systematic error is recorded with every method used, holds [32,33,34,35,36,37,38]. This fact supports the decision at this juncture to define the mean value from all the devices examined as a reference, without to the intention of assigning a favored role for any single procedure.

#### 4.1.4. Causes of Variability in tHb Measurement

The core idea underlying this study is the analysis of the measurement accuracy of methods with a reference character, as well as the inclusion of a clinically widespread system such as the HemoCue [26]. Another focus is on the range from 3–9 g/dL, since this is where clinical decisions are required that will be influenced by measurement accuracy. In order to ensure that these questions are answered with some degree of certainty, the pre-analytical side was methodologically taken out of the equation as far as possible. Furthermore, the entire tHb range of 3–18 g/dL was to be tested safely and equivalently for each method, taking clinical handling into account. These goals have been achieved according to the agreement depicted in Table 3. Nevertheless, it is still necessary to point out any potential causes of variability (Table 5).

The pre-analytical error is one of the main limitations, which needs to be reduced as far as possible when determining the measurement accuracy of the systems under test. The pre-analytical error, i.e., the uncertainty when taking the blood sample from the patient [44], combined with the choice of the sampling site (arterial, venous, or capillary) [15,41,43], leads to systematic errors that would also be included in the statistical analysis.

In order to avoid these distortions in the context of the present study, we chose to take six blood units as our starting point. By randomly assigning these to the test and measurement methods on the basis of the syringes and cuvettes commonly used in clinical practice, it was possible to directly avoid both types of error.

Other causes of error, such as the feeding of the samples, have also been largely avoided by repeating the measurements three times with constant agitation of the samples.

### 4.2. Representation According to B and A

The basic idea—that each method has a small but systematic error—is relevant and pertinent for every measurement method. The statistical processing according to B and A requires systematic conditions and has been regularly questioned [32,33,34,35,36,37,38]. Ultimately, these deliberations have been given broad consideration in the present study in accordance with the current list of criteria [32,33,34,35,36,37,38]. The statistically defined variable “confidence interval” is a common tool, although this refers to the mean of the data, and is retrospectively related to a data set. The same is true for the B and A analysis, but here each data point is taken into account. In order to make a prospective statement about the measurement accuracy of a measured value, two conditions are required: the first is based on each data point, and the second is based on a confidence level in the sense of a prediction.

### 4.3. Overlapping of B and A Definitions with the 95% Prediction Interval

The prediction interval is the most important tool in this analysis. In clinical use, it allows the physician to make a reliable assessment of the measurement accuracy of a hemoglobin value measured on the patient, and can therefore serve as the basis for making a clinical decision. Physicians can use this to directly assign the measured value for tHb obtained from the patient to a predicted value with certain upper and lower limits. In addition, the representation in the form of the absolute differences compensates for any uncertainties between the available methods (Figure 5). Furthermore, the strictly linear curve is essential for the ability of the prediction interval to demonstrate validity for the entire segment below 9 g/dL.

### 4.4. Presentation as TLA

The error grid analysis according to Clark is a form of representation for estimating the measurement accuracy of POCT and POCM devices [16,39] and it also involves the definition of clinically relevant zones as laid out by Zatloukal et al. [47]. However, this form of overview has two major blind spots: firstly, the systematic measurement error of the reference is negated, and secondly, an evaluation of the measurement accuracy in the form of a representative zone is not provided for a range below 6 g/dL. The TLA form of representation was an attempt to close these blind spots [7]. This is based on the B and A principle that every device or process will show some systematic error. This blind spot is eliminated by revealing the systematic differences compared to the mean values. Furthermore, this method adapts the limits when approaching 3 g/dL, taking into account the necessary measurement accuracy at this level. This analytical method had its origins in aviation with the airplane altimeter [39], where the requirement for measurement accuracy increases as the distance to the ground decreases.

### 4.5. Clinical Consequences from the Results

The methods with a reference character in the present study are the CO-oximeters (modules of the BGA devices), the automated hematology analyzers (AHA), and the AHD method. Correspondingly, small but systematic errors are then propagated in the calibration for downstream processes and devices [1,10].

“Inadequate measurement is associated with inappropriate transfusion decisions” is quoted in [14]. The risks for the patient, the reduced availability of RBCs due to a decline in blood donations, and ultimately the costs, are essential factors in PBM. The justification for every transfusion according to the current guidelines must be documented for the reasons mentioned elsewhere [51,52,53,54]. The value for tHb is decisive in this respect [14,51,52].

#### 4.5.1. For Patient Blood Management (PBM)

PBM is an effective tool for ensuring patient safety, adapted to the reduced supply of blood donations, and the general economic requirement to lower the costs of therapy [4,6,55]. The broad common denominator here is the tolerance of significantly lower tHb levels. This in turn leads directly to the underlying question of the present study. POCT and POCM measurements support PBM in everyday clinical practice [14,56]. The main advantage is the rapid and repeated availability, which results in the time window (turnaround time) until the therapeutic decision and implementation being reduced [9,56]. In contrast to the POCT, the POCM is a valuable instrument due to its continuous use and its display as a trend analysis. The relative measurement inaccuracy of such methods can be considered as an error, but this can be corrected, and thus adjusted by measuring a blood sample [14].

#### 4.5.2. For the Identification of a Critical Threshold for Lowest tHb

Where is the pathophysiological threshold of the tHb in relation to an anemia-related morbidity or mortality? In order to try answer this question, we would like to refer here to some statements in various meaningful studies [2,57,58,59]:

Habler et al. [2] stated that in clinical practice, it might be problematic to identify the threshold of the patient’s individual anemia tolerance. The results of patient studies with respect to postoperative anemia and patient mortality may provide an assistance in this respect.

In critically ill cardiac patients, the tHb level at which serious morbidity or mortality may occur is a continued debate and a fixed transfusion trigger will not provide an optimal risk–benefit profile in this population [57].

**Figure 7:**
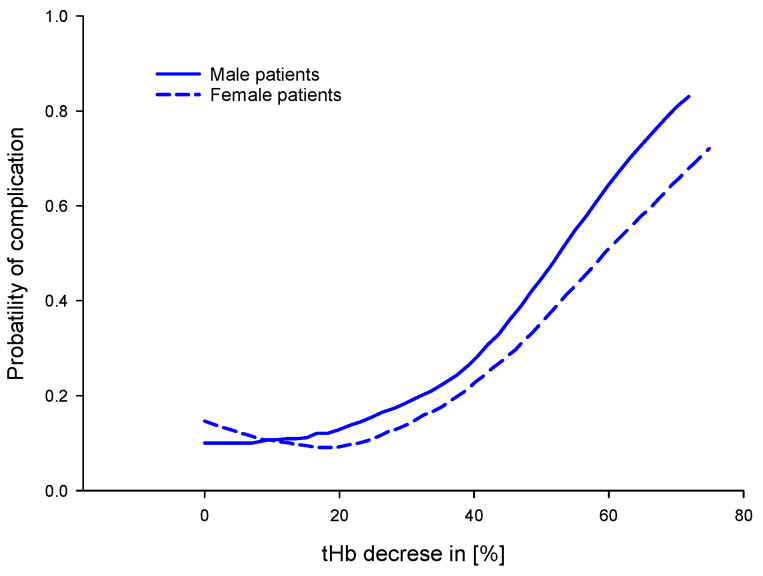
Relationship between decrease in tHb level and morbidity in patients who undergo major gastrointestinal surgery, stratified by sex. Adapted from [58].

Symptoms of anemia and intravascular volume are two variables to consider when determining a threshold for RBC transfusion [58]. Because these are sometimes subjective and difficult to assess, the nadir tHb level has been adopted to guide transfusion practices [58]. The assessment of preoperative factors associated with a decrease in tHb levels may support risk classification in patients who undergo major gastrointestinal surgery [58]. Furthermore, the decrease in tHb level (Figure 7) is also a marker for this risk stratification, demonstrating a correlation between a decrease of 20 to 80 % in tHb level with the probability of complications, where female patients show a small but systematic reduction in complications compared to males [58].

**Figure 8:**
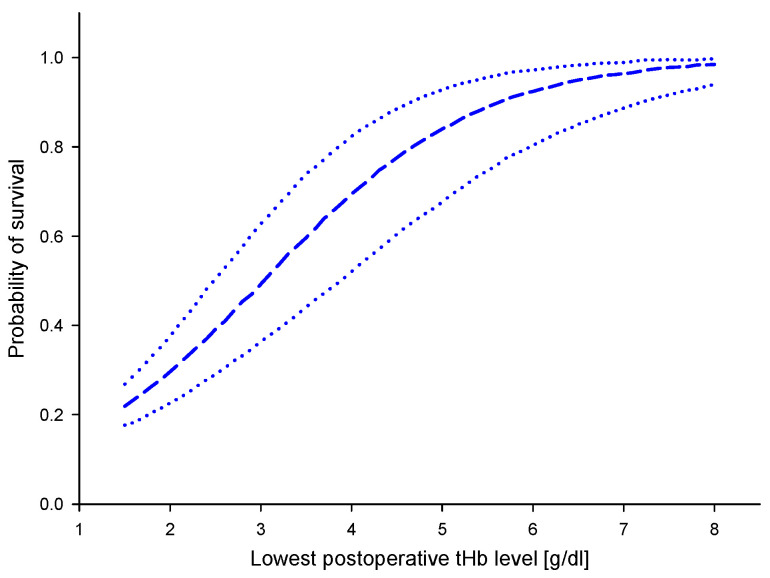
Probability of survival according to lowest postoperative tHb level, based on regression models and simulated data (dashed line), adapted from [59], dotted lines represent upper and lower 95% confidence interval of the pooled data.

The morbidity and mortality in patients with extremely low tHb levels have been assessed by Shander [59]. This study confirmed the risk of severe anemia at nadir tHb levels below 6 g/dL [59]. As such, the question of using tHb levels to decide on transfusion of RBCs can only be looked at critically. However, analysis of pooled data sets might provide better insights in this respect (Figure 8).

The error in tHb measurement accuracy is greatest when compared to the corresponding tHb value at low concentrations. From a clinical perspective, it is precisely here that the most reliable measurement accuracy is needed. To extract this clinically absolutely necessary information from the data, the methods of analysis including “relative differences”, “limits of agreements” according to B and A, the “95% prediction interval”, and the clinical approach of “tolerance level analysis” were selected. These are powerful tools, and the results from these analyses provide the necessary security for clinical consideration.

The 95% prediction interval is the target tool for handling the value of tHb measurement in the clinical context. In this investigation, the limits are +/− 0.3 g/dL. It allows the physician to determine the position with respect to the probability of the survival, as shown in the diagram of Figure 8.

#### 4.5.3. For the Decision-Making Concerning Treatment

“Accurate diagnosis at the individual level is important to identify individuals who require treatment”. This quote from [3] rounds off the objective of this study, namely to establish a basis for quantifying tHb measurement accuracy for reaching a clinical decision, here also specified for the segment from 3 to 9 g/dL tHb. The decision to undertake a blood transfusion depends on the requirements of the immediate situation and individual thresholds. These individual thresholds are often below a hemoglobin concentration of 9 g/dL [53,57,59,60,61] in patients with a restrictive transfusion handling. The statistical tools presented here have provided a solid basis for making safe decisions in the clinical setting, and represent an applicable basis for transfusion decisions in combination with the legal requirements for PBM.

The data and results presented in this investigation implicate two more valuable characteristics with a potential input on decision making: First, they may be embedded in a system with artificial learning for decision making [62]. Second, the data served as reference values with respect to the training of machine learning systems, i.e., regression models [63,64].

In future work, patient demographics, vital signs, or other physiology records could be implemented into such modeling. By combining both real-time monitoring data and patient’s individual records, support may become available for making prompt decisions on blood transfusions [62].

## 5. Conclusions

The value of hemoglobin in its role as a vital carrier of oxygen is indisputable. Causes of error in tHb measurements exist while the implemented practice in patient blood management is increasingly leaning towards tolerance of extremely low tHb concentrations in patients. Crucial decisions concerning patient treatment are based to a large extent on measurement results. All of these facts emphasize the need to focus on the measurement accuracy of the respective devices.

In summary, an extremely low but systematic variability of the devices and methods used for measuring hemoglobin concentrations has been confirmed. Therefore, the tolerance level analysis may be used as a basis when considering measurement accuracy in clinical decisions. The decisive factor for patient blood management in clinical practice is the 95% prediction interval, which is linear in the segment below 9 g/dL tHb at +/− 0.3 g/dL.

## Figures and Tables

**Table 1:** Specifications for the test devices. BGA = blood gas analyzer, n = number of devices or methods, N = number of measurements per sample, POCT = point of care testing, SLS = sodium lauryl sulfate reagent, AHD = alkaline hematin detergent (AHD575), DIN = German Institute for Standardization, * Werfen Group.

Device	Manufacturer	n	Serial Number	N	System	Method	Measurement
GEM 4000	Instrumentation Laboratory *	2	10033403	3	BGA	CO-oximeter	optical
			10043637	3	BGA	CO-oximeter	optical
GEM 5000	Instrumentation Laboratory *	2	16080371	3	BGA	CO-oximeter	optical
			18122404	3	BGA	CO-oximeter	optical
ABL 80 COOX	Radiometer	1	305040	3	Stand-alone	CO-oximeter	optical
OSM 3	Radiometer	1	89R62N03	3	Stand-alone	CO-oximeter	optical
Sysmex XN 9000	Sysmex Europe GmbH	1	12784 + 15591	3	Analyzer	SLS reagent	optical
HemoCue 201^+^	HemoCue AB, Sweden	3	945013284	3	POCT	Microcuvette	optical
			619012266	3	POCT	Microcuvette	optical
			619012265	3	POCT	Microcuvette	optical
Norm	DIN 58931:2010-08	1		3	Laboratory	AHD reagent	optical

**Table 2:** Data processing and assignments to the references (REF I + II).

		All Data		Mean of the Identical Devices	
Devices & Method	Device Nr.	n =	Assigned to	n =	Assigned to
GEM 4000	01	54	REF I	54	REF II
	02	54	REF I		
GEM 5000	01	54	REF I	54	REF II
	02	54	REF I		
ABL 80 CO-OX	01	54	REF I	54	REF II
OSM 3	01	54	REF I		
XN 9000	01	54	REF I	54	REF II
HemCue 201^+^	01	54	REF I		
	02	54	REF I		
	03	54	REF I		
AHD	01	54	REF I		
Total n =		594		216	

**Table 3:** Quality criteria for the test devices. Regression analysis of Deming and Passing–Bablok = PB, RMSE = root mean square error, RSQ = R squared.

Devices	Deming Slope	Deming Intercept	PB Slope	PB Intercept	RMSE	RSQ
Hemocue 1	1.002	−0.321	1	−0.295	0.327	0.999
Hemocue 2	0.983	−0.232	0.983	−0.196	0.434	0.999
Hemocue 3	0.997	−0.246	0.996	−0.2	0.3	0.999
ABL 80	0.987	−0.109	0.986	−0.104	0.266	1
XN 9000	0.992	−0.149	0.993	−0.123	0.264	0.999
GEM 5000 1	1.018	0.066	1.011	0.082	0.357	0.998
GEM 5000 2	1	0.151	0.999	0.115	0.193	0.999
GEM 4000 1	1	0.162	0.996	0.157	0.23	0.999
GEM 4000 2	1.001	0.139	0.998	0.151	0.231	0.999
AHD	1.002	0.423	1.004	0.408	0.46	0.999
OSM 3	1.023	0.064	1.027	0.023	0.357	0.999

**Table 4:** The absolute differences in [g/dL] according to the Bland and Altman procedure with bias (mean), precision (+/− SD), and limits of agreement (+/− 1.96 SD). The relative differences are expressed as percentages compared to the respective target values in [g/dL].

	Absolute Differences in [g/dl]			Relative Differences in [%]		
All Device in Test	Mean	SD	1.96 SD	Mean	SD	1.96 SD
Hemocue—REF I	−0.33	0.14	0.28	−3.93	2.21	4.43
GEM 4000—REF I	0.16	0.18	0.35	2.07	2.29	4.59
GEM 5000—REF I	0.20	0.18	0.36	2.22	1.69	3.38
ABL 80—REF I	−0.24	0.14	0.28	−2.69	1.38	2.77
XN 9000—REF I	−0.23	0.15	0.30	−2.52	1.67	3.34
OSM 3—REF I	0.30	0.21	0.42	2.88	1.97	3.93
AHD—REF I	0.44	0.16	0.33	5.50	3.17	6.35
Clinically relevant devices						
GEM 4000—REF II	0.12	0.11	0.21	1.48	1.37	2.74
GEM 5000—REF II	0.16	0.11	0.23	1.66	0.99	1.98
ABL 80—REF II	−0.28	0.18	0.36	−3.22	1.98	3.97
XN 9000—REF II	−0.27	0.20	0.40	−3.06	1.99	3.98

**Table 5:** List of causes for the variability in tHb measurement.

		References
Origin of the blood sample		
	Capillary vs. arterial vs. venous	[15,41,42,43]
	Population dependent	[3,20,41,42]
Blood collection technique		
	Preanalytical failure	[9,23,42,44]
	Choice of anticoagulants	[23]
Methods of Measurements		
	Standard and reference methods	[36,43,45,46,47]
	Invasive and non-invasive measurements (POCT, POCM)	[1,11,20,46,48,49,50]

## Abbreviations

AHA	Automatic hematology analyzer
AHD	Alkaline hematin detergent
B and A	Bland and Altman
BGA	Blood gas analyzer
DIN	German Institute for Standardization
HiCN	Cyanmethemoglobin method
PB	Passing–Bablok
PBM	Patient blood management
POCM	Point-of-care monitoring
POCT	Point-of-care testing
RBC	Red blood cell
REF	Reference
RMSE	Root mean square error
RSQ	R squared
SD	Standard deviation
SLS	Sodium lauryl sulfate
THb	Total hemoglobin concentration
TLA	Tolerance level analysis
